# MerR and ChrR mediate blue light induced photo-oxidative stress response at the transcriptional level in *Vibrio cholerae*

**DOI:** 10.1038/srep40817

**Published:** 2017-01-18

**Authors:** Mehmet Tardu, Selma Bulut, Ibrahim Halil Kavakli

**Affiliations:** 1 Computational Science and Engineering, Koc University, Rumeli Feneri Yolu, Sariyer, Istanbul, Turkey; 2 Chemical and Biological Engineering, Koc University, Rumeli Feneri Yolu, Sariyer, Istanbul, Turkey; 3 Molecular Biology and Genetics, Koc University, Rumeli Feneri Yolu, Sariyer, Istanbul, Turkey

## Abstract

Blue light (BL) is a major environmental factor that affects the physiology, behavior, and infectivity of bacteria as it contributes to the generation of reactive oxygen species (ROS) while increasing photo-oxidative stress in cells. However, precise photo-oxidative response mechanism in non-phototrophic bacteria is yet to be elucidated. In this study, we investigated the effect of BL in *Vibrio cholerae* by using genetics and transcriptome profiling. Genome-wide analysis revealed that transcription of 6.3% of *V. cholerae* genes were regulated by BL. We further showed that BL enhances ROS production, which is generated through the oxidative phosphorylation. To understand signaling mechanisms, we generated several knockouts and analyzed their transcriptome under BL exposure. Studies with a double-knockout confirm an anti-sigma factor (ChrR) and putative metalloregulatory-like protein (MerR) are responsible for the genome-wide regulation to BL response in *V. cholerae*. Collectively, these results demonstrate that MerR-like proteins, in addition to ChrR, are required for *V. cholerae* to mount an appropriate response against photo-oxidative stress induced by BL. Outside its natural host, *V. cholerae* can survive for extended periods in natural aquatic environments. Therefore, the regulation of light response for *V. cholerae* may be a critical cellular process for its survival in these environments.

Light perception is crucial for the survival of most organisms; it enables them to adjust their physiology and metabolism to the changing environmental conditions. Light, in contrast, can also pose a threat to any living organism due to its deleterious effects on nucleic acids, lipids and proteins^1^. Therefore, the capacity to sense and respond to light is important for prokaryotes and eukaryotes to survive and adapt themselves to the selective pressure of solar irradiation.

In the ultraviolet-visible (UV-VIS) spectrum, only blue light (BL) and UV radiation can reach significant depths in freshwater and marine ecosystems^2^. Therefore, most marine organisms, including non-phototrophic bacteria, have different types of BL photoreceptors such as phototropins, cryptochromes (CRYs), and other proteins containing BLUF (BL using FAD) domains and LOV (Light, Oxygen and Voltage) domains to sense the light^3^,^4^. The LOV- and BLUF-domain-containing proteins absorb BL and initiate the photo-oxidative stress response by regulating the transcription of genes responsible for ROS production in some bacteria^5^,^6^,^7^.

*Vibrio cholerae* O1 biovar El Tor N1696 (hereafter abbreviated as *V. cholerae*) is a Gram-negative facultative human pathogen that colonizes the human intestine. Outside its host, it can survive for extended periods in natural aquatic environments. Therefore, the regulation of light response for *V. cholerae* may be a critical cellular process for its survival. The sequencing of *V. cholerae* genome revealed three *phr* genes that encode photolyase/cryptochrome proteins as the sole BL photoreceptors, indicating that BL may regulate gene expression in this organism^8^,^9^,^10^. Characterization of these *VcPhr* genes displayed that one gene encodes a CPD photolyase (VCA0057) while the other genes encode for CRYs named as *VcCry1* (VC1814) and *VcCry2* (VC1392)^10^. Subsequent studies reported that both VcCRYs are CRY-DASH proteins and have photolyase activity which specifically repair CPD photoproducts in single-stranded DNA (ssDNA). Therefore, they are called as ssDNA photolyases^11^. CRYs and photolyases also regulate other cellular processes in response to BL in organisms ranging from fungi to plants^12^,^13^,^14^,^15^. Therefore, in the present study, we used molecular genetics and transcriptomics approaches to investigate the BL response mechanism in *V. cholerae* and explore how cells produce an appropriate BL response at the genome-wide level.

In this study, RNA-seq analysis indicated that *V. cholerae* responds to BL by regulating the transcript levels of 6.3% of its total genes. Further study enabled us to identify that BL causes the photo-oxidative stress by inducing ROS production. Treatment of the cells with the uncoupling reagents 2,4-dinitrophenol and flufenamic acid revealed that BL exposure results in ROS production through the electron transport chain (ETC). Further inhibition studies using rotenone and malonate indicated that the source of ROS production is complex II (succinate dehydrogenase) within ETC. To identify how ROS mediates the photo-oxidative stress response, we generated knockout cell lines by deleting the candidate genes that may play a role in transmitting the effect of increased ROS level. Genome-wide studies of the knockout cell lines indicated that both an anti-sigma factor (ChrR, VC2301) and a putative metalloregulatory-like protein (MerR, VCA0056) mediate the effect of ROS to control the genome-wide gene expression in *V. cholerae*. Analyses of differentially expressed genes (DEGs) showed that BL strongly affects the transcription of genes related to cellular protection, carbon metabolism and DNA repair.

## Results and Discussion

To identify the pathways affected solely by BL in *V. cholerae*, wild-type and knockout cells were irradiated with 50 μmoles m^−2^s^−1^ BL as previously described^16^. Total RNA from dark- and BL-treated cells were isolated followed by tRNA and rRNA depletion and library preparation. The quality of the library was assessed by BioAnalyzer 2100 and then the samples were sequenced using Illumina MiSeq platform.

### Mapping and coverage of RNA-seq data

The bacterial strains and plasmids used in this study are listed in Table 1. After sequencing and de-multiplexing, RNA-seq data were aligned to the *V. cholerae* reference genome^17^ and gene expression values were calculated using Rockhopper^18^. An overview of the sequencing and mapping data for wild-type and mutant cells is shown in Supplementary Table S1. Supplementary Table S2 summarizes the RNA-seq gene expression data across all samples. To evaluate reproducibility among biological replicates, a Pearson’s correlation test was performed on the expression values. There was a strong correlation between biological replicates for each condition based on the calculated Pearson’s correlation coefficients (R^2^ > 0.95) (Supplementary Fig. S1). This finding confirmed that there was consensus among the replicates in each condition, which allowed us to perform further differential gene expression analyses.

To identify differentially expressed genes (DEGs) in response to BL and their operon organization, we calculated the difference in the number of mapped Reads Per Kilobase of exon per Million mapped reads (RPKM) between dark- and BL-treated samples using Rockhopper. In total, 222 (6.3%) DEGs were identified with |log_2_ fold change| ≥ 1 and a false discovery rate (FDR) ≤ 0.01, in response to BL (Supplementary Table S3). Of those genes, 81 genes were down-regulated and 141 genes were up-regulated. Further analysis of the 222 DEGs (designated as Set1) revealed that 117 of them were grouped under 57 predicted operons (Supplementary Table S4) while 105 DEGs were not grouped under predicted operons.

### Validation of DEGs using quantitative real-time PCR under blue light versus dark conditions

A total of 21 representative up- and down-regulated DEGs (VC0837, VC0943, VC1118, VC1248, VC1263, VC1359, VC1392, VC1484, VC1570, VC1643, VC1814, VC1922, VC2088, VC2301, VCA0055, VCA0615, VCA0782, VCA0798, VCA0809, VCA0957, VCA1087), designated as Set2 DEGs, were selected to validate the RNA-seq results. Among the selected DEGs, 12 were from 12 different operons while nine were not grouped into operons. Cells were exposed to BL, and total RNA was isolated from each sample. After conversion of the total RNA into cDNA, real-time PCR (qRT-PCR) was performed using appropriate primers. A comparison of qRT-PCR results (relative changes in the transcription level of DEGs from BL exposed cells were calculated with respect to dark condition) and RNA-seq results revealed similar expression patterns for each gene, indicating that the RNA-seq results were reliable (Supplementary Fig. S2).

To determine whether such genome-wide regulation in *V. cholerae* is specific to BL, cells were also grown under red light (RL) condition. After preparation of cDNAs from RL- and dark-treated cells, the transcription levels of Set2 DEGs were measured by qRT-PCR. As shown in Supplementary Fig. S3, RL did not significantly affect the transcription levels of Set2 DEGs, suggesting that these DEGs resulted specifically from exposure to BL.

### Blue light regulated pathways as determined by Gene Ontology (GO) and Kyoto Encyclopedia of Genes and Genomes (KEGG) pathway enrichment analyses

To functionally categorize DEGs in cells exposed to BL, a GO term enrichment analysis was performed using the PANTHER classification tool^19^. The assigned GO terms were used to classify functions of the DEGs based on biological processes, molecular functions, and cellular components.

**Figure 1:**
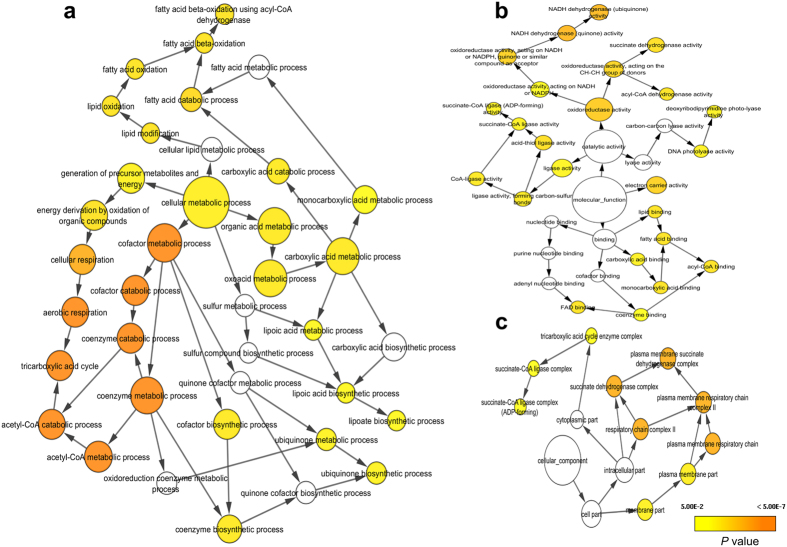
Gene Ontology (GO) term network analysis of all differentially expressed genes (Set1 DEGs). Functional enrichment analysis was performed for all DEGs with the BINGO plug-in in Cytoscape. Assigned GO terms were used to classify functions of DEGs based on **(a)** biological processes, **(b)** molecular functions, and **(c)** cellular components. The yellow and orange nodes represent terms with significant enrichment, with darker orange representing a higher degree of significance, as shown by the legend on graph. White nodes are terms with no significant enrichment, but are included because they have a significant child term.

To define the biological functions of Set1 DEGs, GO and KEGG analyses were carried out. Forty-five significantly enriched GO terms (*p* < 0.05) were identified, including single-organism metabolic process (61 genes), oxidation-reduction process (32 genes), metabolic process (83 genes), cellular respiration (13 genes), aerobic respiration (10 genes), tricarboxylic acid cycle (nine genes), carboxylic acid metabolic process (27 genes), single-organism process (72 genes), chemotaxis (nine genes), response to chemical (10 genes), catalytic activity (70 genes), DNA photolyase activity (four genes), and plasma membrane (21 genes). A detailed table of all GO terms is provided in Supplementary Table S5. Then, a GO term network was constructed using Cytoscape with the BINGO plug-in ref. 20. As shown in the results of the GO term network analysis in Fig. 1a, metabolic processes were significantly (*p* < 0.05) linked to cellular respiration, fatty acid oxidation, cofactor metabolic processes, and organic acid metabolic processes. Additionally, a molecular function network analysis indicated that genes related to FAD binding, DNA repair, oxido-reductase activity, and electron carrier activity were significantly (*p* < 0.05) linked (Fig. 1b). Further analysis with respect to cellular components revealed that the respiratory chain complex, plasma membrane, and succinate dehydrogenase complex play a significant (*p* < 0.05) role in the BL response of *V. cholerae* (Fig. 1c).

After the GO term analysis of the Set1 DEGs, a KEGG pathway enrichment analysis was performed to identify the metabolic or signal transduction pathways that were highly regulated under BL. Of the Set1 DEGs, 145 were assigned to 14 significantly enriched pathways (Table 2) and the remaining DEGs were categorized as hypothetical genes. The following pathways were found to be significantly (*p* < 0.05) up-regulated under BL: citrate cycle (TCA cycle), butanoate metabolism, geraniol degradation, C5-branched dibasic acid metabolism, propanoate metabolism, lysine degradation, oxidative phosphorylation, carbon metabolism, fatty acid degradation, and biosynthesis of secondary metabolites. The following pathways were found to be significantly (*p* < 0.05) down-regulated under BL: bacterial chemotaxis, two-component system, glyoxylate and dicarboxylate metabolism, and ABC transporters. Since most of the GO and KEGG assignments and distributions were related to energy metabolism, biosynthesis of secondary metabolites, DNA repair, and bacterial chemotaxis; our results indicate that the DEGs were involved in a wide range of regulatory functions in *V. cholerae*.

Among the Set1 DEGs, 77 were categorized as hypothetical proteins in the current KEGG database, highlighting that our understanding of the molecular mechanism of the BL response in *V. cholerae* is incomplete. We used the Annocript pipeline^21^ to annotate these 77 hypothetical DEGs. Annocript successfully annotated 29 DEGs, based on the data at the Swiss-Prot (SP) and UniRef90 databases^22^ (Supplementary Table S6).

### Investigating the role of cryptochrome in blue light response

**Figure 2:**
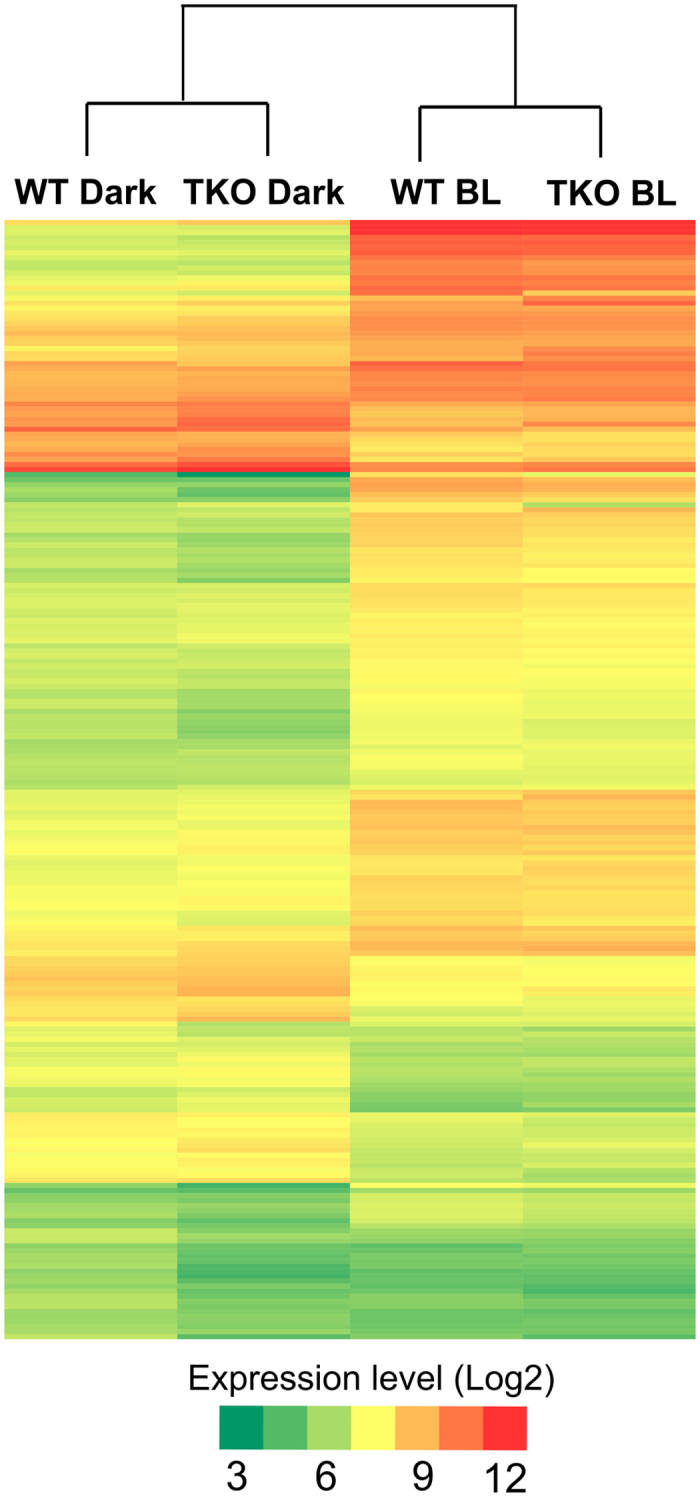
Heatmap display of expression values of differentially expressed genes in wild-type and Δ*cry1*Δ*cry2*Δ*phr* knockout (TKO) cells. Expression levels are represented by color: green, lowest expression level; yellow, moderate expression level; red, highest expression level. Extreme values in color gradient are 3 to 12 in log_2_ scale.

DEG analysis showed that operons containing genes (VC1392, VC1814 and VCA0057) in cryptochrome/photolyase family (CPF) members were highly up- regulated by BL exposure. Previous studies have shown that both cryptochrome and photolyase regulate several genes in response to BL in organisms ranging from fungi to plants^12^,^13^,^14^,^15^,^23^. This raises possibility of CPF involvement in BL reception and genome-wide regulation in *V. cholerae*. Therefore, we generated a triple-knockout mutant (Δ*cry1*Δ*cry2*Δ*phr*) to investigate the role of CPF members in BL regulation. The triple mutant cells were exposed to BL, and then RNA-seq was performed. Analysis of RNA-seq data from wild-type and triple mutant cells revealed that the same genes (Set1 DEGs) were up- and down-regulated under both dark and BL conditions, as shown in the heat map (Fig. 2). This result indicated that previously identified CPF genes^10^ are not involved in BL-mediated gene expression in *V. cholerae*. To investigate whether this organism possesses any type of photoreceptors other than CPF members, we performed a domain composition analysis of 3826 *V. cholerae* proteins in the Conserved Domains Database with Rpsblast (e-value ≤ 10^−5^). Our analysis revealed that there are no other kinds of photoreceptors (Supplementary Table S7), which indicates that some kind of pigment(s) or a low conserved BL photoreceptor(s) may be involved in BL reception in this organism.

### Blue light induces genome-wide transcriptional regulation due to photo-oxidative stress

**Figure 3:**
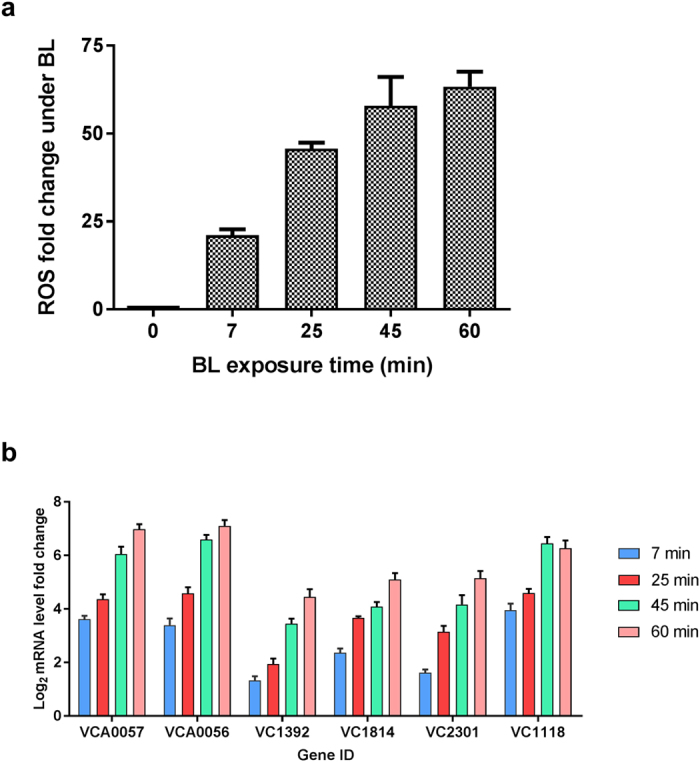
Time-dependent reactive oxygen species (ROS) accumulation and qRT-PCR measurement after blue light (BL) exposure. **(a)** Samples were taken at 7, 25, 45, and 60 min after BL exposure and total ROS amount were measured using 2′,7′-dichlorofluorescin diacetate (DCF-DA). Fold change was calculated between dark- and BL-treated samples at indicated times. **(b)** qRT-PCR measurement of transcription level of several genes selected from Set1 DEGs with respect to BL exposure time. Each colored bars with standard errors represent relative mRNA levels of genes at indicated BL exposure time with respect to dark conditions (log_2_ fold) determined from three independent biological replicates (*n* = 6).

Various environmental stimuli, including high-fluence BL, result in ROS generation. These molecules can act as signaling molecules to regulate a number of developmental processes and stress responses in bacteria^24^,^25^,^26^. We performed a series of experiments to determine whether *V. cholerae* produces ROS under exposure to BL. Cells were illuminated with blue, red and yellow lights for various time periods, and total ROS were measured using the fluorogenic dye 2′,7′ dichlorofluorescein diacetate (DCF-DA). There was a gradual increase in intracellular ROS levels over time only in the presence of the BL, and ROS production was saturated after 45 min of BL exposure (Fig. 3a). We further investigated the effect of time-dependent ROS formation on the expression levels of selected DEGs from Set1 by qRT-PCR. Analysis of the data indicated that ROS measurements (Fig. 3a) and qRT-PCR results (Fig. 3b) were correlated. Collectively, these results showed that BL induced the formation of ROS, which caused oxidative stress in *V. cholerae.* This phenomenon, the so-called photo-oxidative stress response, has been observed in many different organisms including phototrophic and non-phototrophic bacteria^26^,^27^ such as *Rhodobacter sphaeroides*^24^, *Myxococcus xanthus*^28^, *Pseudomanas aeruginosa*^29^, and *Caulobacter crescentus*^30^.

Analysis of the Set1 DEGs indicated that the genes induced by photo-oxidative stress in this organism encode proteins with protective and repair functions. In the cell, ROS can affect membrane lipids, proteins, and nucleic acids^31^. Lipid peroxidation by ROS leads to loss of cell integrity^32^ and cell leakage, which, in turn, affects essential cell membrane processes such as transport and energy generation. In *V. cholerae*, a putative gene (VC1122) encoding cyclopropane-fatty-acyl-phospholipid synthase (CFA synthase) was substantially up-regulated upon exposure to BL. This enzyme catalyzes the cyclopropane ring formation of bacterial phospholipids using *S*-adenosylmethionine as the substrate. It has been shown that modification of the membrane by CFA synthase protects the cell against ROS and thereby minimizes its susceptibility to further damage^33^,^34^. It is possible that the putative CFA synthase modifies the plasma membrane to protect the cell against the harmful effects of ROS in *V. cholerae*.

Since the presence of BL is an indicator of UV-light, *V. cholerae* may use photo-oxidative stress mechanism induced by BL to avoid the harmful effects of UV-light via increasing the transcript levels of *phr* genes, whose products are involved in genome repair. Such regulation has been demonstrated in other organisms as well^13^,^14^. Two hypothetical genes (annotated by Annocript) encode putative glutaredoxin (VC2044) and glutathione S-transferase omega (VC1096) proteins, which protect cells from photo-oxidative stress. In addition, the transcript level of the gene encoding thioredoxin-dependent thiol peroxidase (VC2160) was also up-regulated in *V. cholerae* by BL. ROS is known to oxidize thiol-containing proteins and macromolecules; therefore, cellular redox systems enable microorganisms to reverse such oxidative damage by the activities of thioredoxins and glutaredoxins^35^,^36^.

Taken together, these results indicate that the photo-oxidative stress generated in response to BL enables *V. cholerae* to regulate the transcription of genes related to cellular protection and DNA repair. Since the ROS assay used in this study measures all types of ROS, further experiments are needed to identify which type of ROS is causing the photo-oxidative stress in this organism.

### ChrR and SigmaE regulate gene expression in a blue light dependent manner

**Figure 4:**
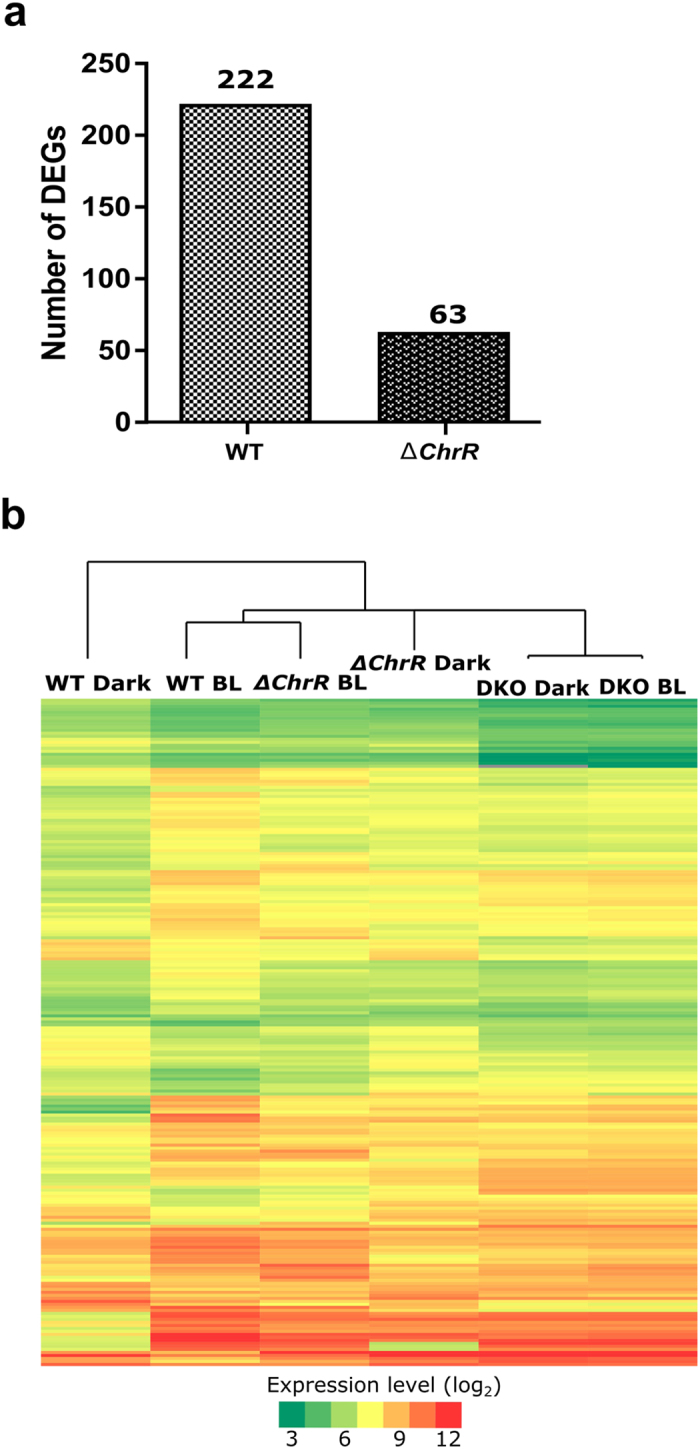
Differentially expressed genes (DEGs) in wild-type (WT), Δ*ChrR*, and Δ*ChrR*Δ*MerR* knockout (DKO) cells under dark and blue-light conditions. (**a**) Pairwise comparison of number of DEGs in WT andΔ*ChrR* cells. (**b**) Heat maps of the Δ*ChrR and DKO* cells were constructed based on Set1 genes (DEGs in WT). Expression levels are represented by color: green, lowest expression level; yellow, moderate expression level; red, highest expression level. Extreme values in color gradient are 3 to 12 in log_2_ scale.

To identify the genes mediating BL regulation in *V. cholerae* at the genome-wide level, we analyzed the operons that responded most strongly to BL. Studies on *R. sphaeroides*^24^ and *C. crescentus*^30^ have indicated that the effect of light-generated ROS is mediated by anti-sigma ChrR and its cognate partner SigmaE (σ^E^)^37^,^38^, and genome-wide regulation of specific genes occurs in a BL-dependent manner. We identified a highly up-regulated putative ChrR operon consisting of *ChrR* (VC2301), *SigmaE* (VC2302) and a hypothetical gene (VC2303). To investigate the role of ChrR in the photo-oxidative response, we generated a Δ*ChrR V. cholerae* mutant. Dark- and BL-treated Δ*ChrR* cells were subjected to RNA-seq to identify DEGs. Analysis of the RNA-seq results indicated that 159 DEGs (Set3 DEGs) out of the Set1 DEGs identified in wild-type cells were no longer regulated in the Δ*ChrR* mutant, while the remaining 63 of the Set1 DEGs were still up- and down-regulated (Fig. 4a). In the Δ*ChrR* mutant, the mRNA expression levels of these Set3 DEGs were elevated in the dark, so their expression level did not change by BL exposure (wild-type BL *vs.* Δ*ChrR* dark in Fig. 4b). This finding indicated that the *ChrR* gene suppresses the transcription of Set3 DEGs.

**Figure 5:**
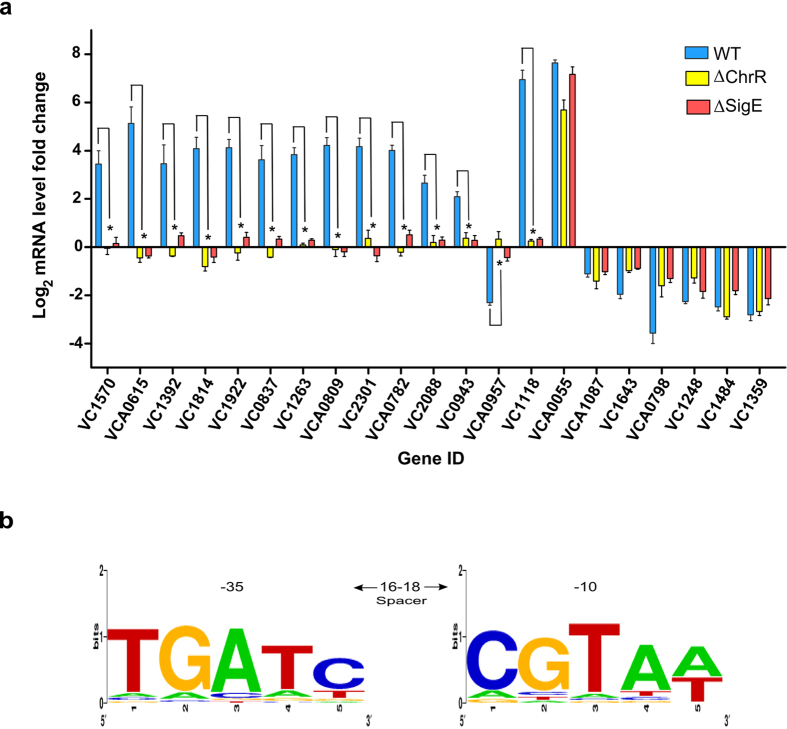
Effect of blue light (BL) on wild-type, Δ*ChrR,* and Δ*SigE* cells. **(a)** Transcript levels of selected 21 DEGs were determined after exposing cells to BL. Blue bars (fold change in wild-type cells), green bars (fold change in Δ*ChrR* mutant) and red bars (fold change in Δ*SigE* mutant) with standard errors represent relative mRNA expression levels with respect to dark conditions (log_2_ fold) determined by qRT-PCR from three independent biological replicates. *n* = 6, *p < 0.05, Student’s t-test. **(b)** Putative σ^E^-dependent promoter motif identified in upstream region of genes controlled by σ^E^. Sequence of σ^E^-binding motif (TGATC-N16-18-CGTAW, where W is A or T) derived from upstream of *SigE* (VC2302) was used to search upstream regions (−300 to +5 relative to predicted translation start site, +1) of all putative σ^E^-dependent genes, allowing for two substitutions, using ‘dna pattern’ tool at RSA website (http://rsat.ulb.ac.be/rsat). Co-ordinate represents the position of 3′ end nucleotide of putative σ^E^-binding motif relative to translation start site (+1).

A number of studies have shown that ChrR and σ^E^ work together to regulate the genome-wide response to BL in various organisms^26^,^39^,^40^. Since we observed that some DEGs were controlled by ChrR, we investigated whether it works with its cognate partner σ^E^, whose transcription is controlled by the same operon. We therefore generated the Δ*SigmaE V. cholerae* mutant and exposed it to BL. We prepared cDNA from the total RNA and measured the transcript levels of Set2 DEGs by qRT-PCR. As can be seen in Fig. 5a, 14 out of 21 genes from Set2 DEGs were not regulated in the Δ*ChrR* mutant. Like in Δ*ChrR* cells, same genes in Δ*SigmaE* cells were not differentially regulated in response to BL. Additionally, the transcript levels of those 14 genes from the Set2 DEGs in BL-treated Δ*SigmaE* cells were similar to those in dark-treated wild-type cells. These results indicated that ChrR and σ^E^ work together in the response to BL, where σ^E^ activates the transcription of the Set3 DEGs. All these findings showed that in the dark, ChrR binds to and suppresses σ^E^.

Upon exposure of cells to BL, σ^E^ is released from its cognate partner ChrR, and it binds to either the operon or to the promoter regions of genes regulated by photo-oxidative stress, as shown in other organisms^24^,^28^,^29^. We also analyzed the upstream regions of the Set3 DEGs whose transcriptional regulation depended on ChrR and σ^E^ by using the ‘dna pattern’ tool at the RSA website (http://rsat.ulb.ac.be/rsat) to find consensus σ^E^-binding sequences^41^. The following promoter sequence recognizable by the σ^E^ factor was deduced: TGATC-N18-CGTAT^42^. This consensus sequence was found (Fig. 5b) in the upstream of 31 operons and 29 genes of the Set3 DEGs (Supplementary Table S8).

A KEGG pathway enrichment analysis was carried out to identify the metabolic pathways of genes that were strongly regulated in response to BL in the Δ*ChrR* mutant. Out of 63 DEGs, 22 were assigned to 11 significantly enriched pathways and 18 were categorized as hypothetical genes. Comparison of the affected pathways between the Δ*ChrR* mutant and wild-type cells revealed that geraniol degradation, fatty acid degradation, propanoate metabolism, and butanoate metabolism pathways (Table 3) were still significantly regulated in response to BL in the Δ*ChrR* mutant.

### Blind phenotype of Δ*ChrR*Δ*MerR* double-knockout mutant in blue light response

As mentioned above, the RNA-seq data from the ΔC*hrR* mutant cells indicated that an additional gene was responsible for regulating 63 genes under BL condition (Fig. 4a). To identify this gene, we examined the transcript levels of the DEGs in Δ*ChrR* cells. We found that the transcriptional levels of the genes, *MerR-*like (VCA0056), *phr* (VCA0057) and a hypothetical gene (VCA0058) under the control of a predicted operon, were highly up-regulated. Proteins from the MerR family of transcriptional regulators (originally described as proteins involved in mercury resistance) are known to mediate light-induced carotenoid synthesis in both *Streptomyces coelicolor* and in *M. xanthus*^43^,^44^. To investigate the possible role of MerR in the BL-induced photo-oxidative stress in *V. cholerae,* we generated a Δ*ChrR*Δ*MerR* double-knockout mutant and performed RNA-seq analysis under both dark and BL conditions. After analyzing DEGs in the double-knockout cells, we observed a light-blind phenotype (Fig. 4b). This result clearly indicated that putative MerR-like protein in *V. cholerae* is required to mediate the effect of BL-induced photo-oxidative stress.

We further analyzed the RNA-seq data from the double-knockout cells to understand how MerR-like protein mediates the transcriptional regulation of DEGs. The transcript levels of some of the DEGs were comparable to those in dark-treated wild-type cells (Fig. 4b), while other DEGs were comparable to those in BL-exposed wild-type cells. These data suggested that MerR may act as a suppressor or an enhancer of those 63 genes. Since the Set3 DEGs levels were comparable between wild type BL-treated and Δ*ChrR* dark-treated cells, we further verified that ROS was still produced in these mutants. As shown in Supplementary Fig. S4, ROS were produced in comparable amounts in all the mutants. These results suggested that ChrR and MerR together or separately regulate gene expression in response to photo-oxidative stress by BL-generated ROS.

### Blue light prompts *V. cholerae* to produce ROS by oxidative phosphorylation

Both GO and KEGG pathway analyses of the Set1 DEGs in wild-type cells provided insights into the main biological processes and pathways related to catabolic reactions. Among these pathways, “propanoate metabolism” and “fatty acid degradation” catabolize imported nutrients into products that are the initial substrates for “citrate cycle (TCA cycle)”, “butanoate metabolism” and “C5-Branched dibasic acid metabolism”. These catabolic pathways provide NADH and FADH_2_ as substrates for the oxidative phosphorylation (electron transport) pathway that produces ATP under aerobic conditions^45^. These results indicate that BL prompts *V. cholerae* to produce energy by cellular respiration.

**Figure 6:**
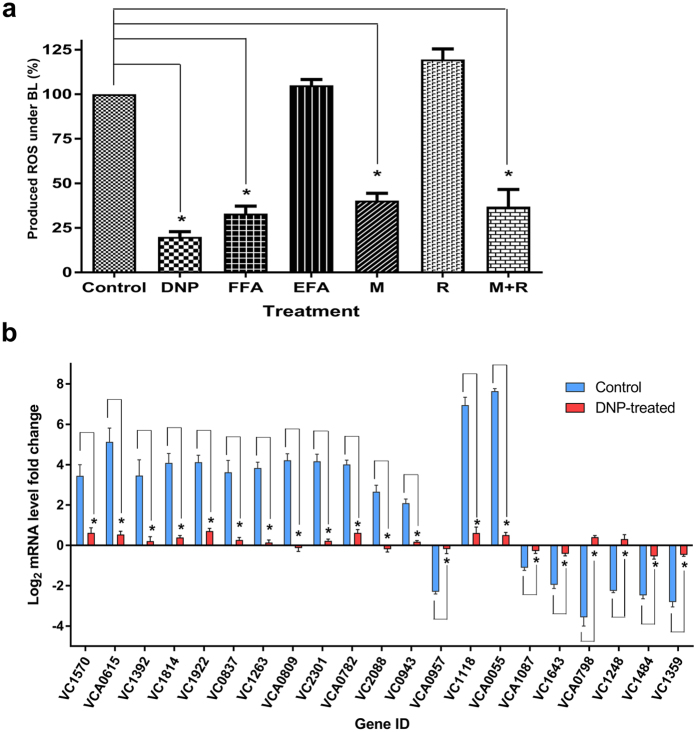
Reactive oxygen species (ROS) accumulation under blue light (BL) after chemical treatments and expression analysis of 2,4-dinitrophenol (DNP)-treated wild-type cells. **(a)** Wild-type cells were treated with 20 mM malonate (M), 100 μM rotenone (R), 500 μM DNP, 50 μM FFA, and 50 μM etofenamate (EFA, a derivative of FFA) for 60 min in darkness, then cells were exposed to BL (50 μmoles m^−2^s^−1^) for 45 min. Fold change was calculated between dark- and BL- treated samples, and then percentage change for each treatment was calculated after setting amount of ROS in wild-type cells at 100%. Error bar: SD; *n* = 6, *p < 0.05, Student’s t-test. **(b)** Transcript levels of selected DEGs were quantified by qRT-PCR after wild-type cells were treated with DNP and exposed to BL. Blue (non-treated wild-type cells) and red (DNP-treated wild-type cells) bars with standard errors represent relative mRNA expression levels with respect to dark conditions (log_2_ fold) determined by qRT-PCR from three independent biological replicates. *n* = 6, *p < 0.05, Student’s t-test.

Several studies have shown that the oxidative phosphorylation pathway is the major source of ROS produced in various organisms^27^,^46^,^47^. Therefore, we hypothesized that ROS production was initiated from oxidative phosphorylation upon exposure of *V. cholerae* cells to BL. To investigate this hypothesis, we treated cells with the uncoupling reagents 2,4-dinitrophenol (DNP) and flufenamic acid (FFA), protonophores that decouple oxidative phosphorylation and result in decreased total ROS production^48^,^49^,^50^. As a control, we also treated cells with a derivative of FFA called etofenamate (EFA) which doesn’t act as an uncoupler of the oxidative phosphorylation. The cells were treated with each molecule in the presence and the absence of BL, and then total ROS production was measured. As seen in Fig. 6a, ROS production was significantly (*p* < 0.05) lower in both DNP- and FFA-treated cells compared to untreated control and EFA-treated cells after BL exposure. We conducted qRT-PCR analyses to quantify the transcript levels of Set2 DEGs in the DNP-treated cells. The transcript levels of the Set2 DEGs differed significantly after BL exposure between the untreated and DNP-treated cells (Fig. 6b). These results revealed that BL causes ROS production through oxidative phosphorylation and results in genome-wide transcriptional regulation in *V. cholerae*.

In addition, analyses of Set1 DEGs and the GO term network revealed that genes encoding components of complex I and II were highly up-regulated under BL. The transcript levels of genes encoding all six subunits (components of the complex I) of Na^+^-translocating NADH-quinone reductase (Na^+^-NQR) (VC2290-VC2295) were up-regulated in response to BL. A previous study showed that Na^+^-NQR represents a major source of extracellular ^1^O_2_ production in *V. cholerae* cells^51^. Also, BL exposure resulted in increased transcript levels of genes encoding the succinate dehydrogenase iron–sulfur subunit (VC2088), the succinate dehydrogenase flavoprotein subunit (VC2089), the succinate dehydrogenase hydrophobic membrane anchor protein (VC2090), and the succinate dehydrogenase cytochrome b556 large membrane subunit (VC2091). Succinate dehydrogenase (complex II) is a flavin-containing enzyme that functions in the TCA cycle as well as in complex II of the ETC. It catalyzes the oxidation of succinate to fumarate and the reduction of ubiquinone to ubiquinol, thereby linking the TCA cycle to the ETC^52^. This protein was shown to be the major source of ROS production resulting in oxidative stress^27^.

**Figure 7:**
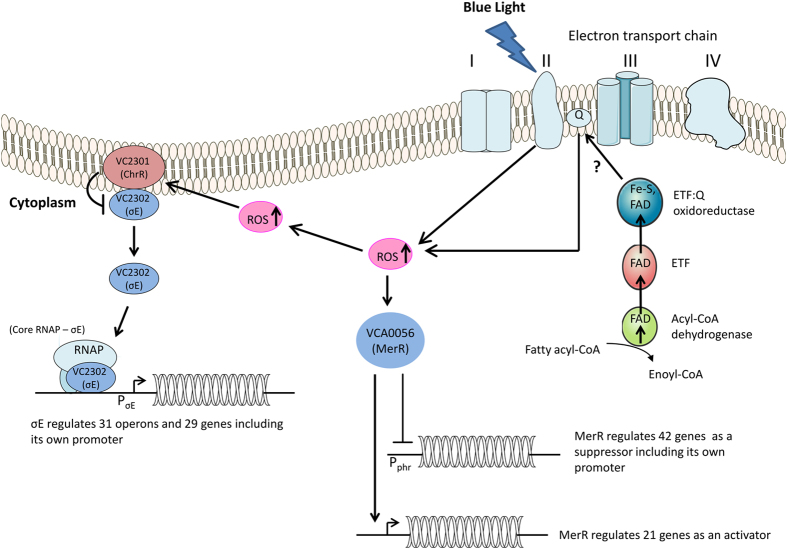
Model summarizing blue-light-mediated gene regulation pathway in *Vibrio cholerae*. Blue light (BL) excites succinate dehydrogenase (complex II) in electron transport chain, which interacts with molecular oxygen to generate ROS. ROS inactivates ChrR, the anti-σ^E^ factor. Dissociated σ^E^ associates with core RNAP, and corresponding RNAP holoenzyme activates Pσ^E^, promoter of the regulatory ChrR-σ^E^ operon. σ^E^ regulates expression levels of 159 genes. Also, ROS interacts with MerR, and suppresses or enhances expression levels of 63 genes includes its own gene. Ovals depict protein factors, short arrows show genes or operons, solid arrows and blunt-ended lines indicate positive and negative regulation, respectively.

To identify the source of BL-induced ROS production, various components of the respiratory chain were inhibited. Rotenone inhibits electron transfer (taken from NADH) from the Fe-S center of complex I (NADH dehydrogenase complex) to ubiquinone, whereas malonate inhibits electron transfer (taken from FADH_2_) from complex II (succinate dehydrogenase complex) to ubiquinone. Therefore, after cells were treated with rotenone or malonate, ROS production was measured under both dark and BL conditions. As seen in Fig. 6a, ROS production was reduced by 50% in the malonate-treated cells, while ROS production in the rotenone-treated cells was comparable to that in control cells in response to BL. However, ROS was still produced in the malonate-treated cells, implying that there are electron inputs from other proteins to the respiratory chain. The Set1 DEGs and GO term network analyses indicated that genes encoding acyl CoA dehydrogenase (VC1740 and VC2231), which participates in β-oxidation of fatty acids, were up-regulated under BL. Acyl-CoA dehydrogenase produces FADH_2_, and then electrons from FADH_2_ are transferred to ubiquinone, the site of ROS production by Q-oxidoreductase (Fig. 7)^27^. Therefore, it is possible that the remaining 50% of ROS were produced by ubiquinone after β-oxidation of fatty acids.

## Conclusion

*V. cholerae* is an enteric bacterium and is therefore insulated from light in its host. However, when it is in its natural aquatic environment or is being transmitted through water and foodstuffs to its host, it may be exposed to sunlight which contains photoreactivating near UV-VIS light and harmful UV-light^10^,^11^. Light is important for non-phototrophic organisms to regulate their cellular signaling and pigment biosynthesis pathways, biofilm formation, and pathogenesis^25^,^44^,^53^. To explore the effect of BL on *V. cholerae* at the genome-wide level, we exposed cells to BL and then conducted RNA-seq analyses. After observing a global response to BL (Fig. 2 and Supplementary Table S3), we decided to identify the mechanism that enables *V. cholerae* to produce such a response. Our studies with wild-type and mutant cells revealed that the cells produce ROS upon exposure to BL (Fig. 3a) and ROS effect is mediated by the MerR-like protein in addition to ChrR-σ^E^ transcription complex (Supplementary Fig. S4). To identify the source of ROS production, we carried out a series of experiments on the ETC under BL exposure. Studies with uncouplers DNP and FFA suggested that ROS is produced through ETC (Fig. 6). Further studies with ETC complex inhibitors suggested that complex II contributes to 50% of the total ROS formation (Fig. 6a) while the remaining %50 of the ROS may originate from quinol or from other sources, which need to be investigated further.

We finally propose a working model for this organism based on our findings (Fig. 7). ROS, generated from ETC, may cause the dissociation of σ^E^ from ChrR. The dissociated σ^E^ binds to the Pσ^E^ region and recruits RNA polymerase to initiate the transcription of 159 genes (Figs 4a and 6). The presence of 63 DEGs out of 222 in Δ*ChrR* cell line (Fig. 4a) suggests that other proteins are also required for the BL response in this organism. Furthermore, our data suggested that a MerR-like protein participates in BL response in this organism. In this effort, we generated a double-knockout cell line, Δ*ChrR*Δ*MerR*, and performed RNA-seq under both BL and dark conditions. RNA-seq results revealed a BL-blind phenotype, in which there were no detectable DEGs in double-knockout cells (Fig. 4b). RNA-seq results from the double-knockout mutant indicated that MerR can act as a suppressor or enhancer to regulate the expression levels of 63 genes, including its own encoding gene (Fig. 4b). Additional study is needed to elucidate whether ChrR and MerR-like proteins work in the same or separate pathways.

## Materials and Methods

### Growth conditions and light experiments

Cultures of *V. cholerae* and *Escherichia coli* were grown in Luria–Bertani (LB) broth (1% tryptone, 0.5% yeast extract, and 1% NaCl) at 37 °C with agitation (250 rpm). Antibiotics were added at the following concentrations unless otherwise noted: streptomycin, 100 μg/ml, ampicillin, 100 μg/ml, and kanamycin, 50 μg/ml. For blue light (BL) experiments, *V. cholerae* cells were inoculated into 5 mL streptomycin-supplemented LB and grown overnight in complete darkness. Then, the dark-grown cells were diluted (1:50) in 15 mL streptomycin-supplemented LB and grown until OD_600_ = 0.8–1.0. After that, the cells were harvested by centrifugation at 7000 xg for 5 min, washed once with PBS and then resuspended in 15 mL PBS buffer. The reason of using PBS is to eliminate growth media that can interfere with light absorption and can cause uneven distribution of the light between the cells. Cells were then exposed to BL (50 μmoles m^−2^s^−1^, fluorescent black light source, each 20 W; General Electric, Cleveland, OH, USA) for 45 min. BL intensity was measured by a UVX Digital Radiometer (UVP, San Gabriel, CA, USA) at 365 nm. To eliminate the presence of the UV-light, a glass piece was used between the samples and black light source. Further, UVX Digital Radiometer (UVP, San Gabriel, CA, USA at 254 nm) was used to measure the UV-light under glass. When we measured no UV-light, cells were treated with BL and then samples were collected for further studies. For red light (RL) experiments, dark-kept cells were exposed to red light (50 μmoles m^−2^s^−1^, red LED light source P10, Hangzhou Zhejiang, China) for 45 min. RL intensity was measured by the Thorlabs PM100 console system (Thorlabs, Newton, NJ, USA) at 630 nm.

### Assessment of photo-oxidative stress

The fluorogenic dye 2′,7′ dichlorofluorescein diacetate (DCF-DA) was used to detect total ROS production. After DCF-DA diffuses into the cell, it is deacetylated by cellular esterases into a non-fluorescent compound that is subsequently oxidized by ROS into 2′,7′ dichlorofluorescein (DCF)^17^. This ROS measurement method is summarized in Supplementary Fig. S5. Briefly, a cell culture grown overnight in the dark was diluted (1:50) with 15 mL streptomycin-supplemented LB and grown until OD_600_ = 0.8. The cells were harvested by centrifugation at 7000 xg for 5 min, washed once with PBS, and then resuspended in 15 mL PBS buffer. Cells were then incubated with 10 μM DCF-DA in the dark for 60 min and then exposed to BL (50 μmoles m^−2^s^−1^) for 45 min. After BL exposure, DCF fluorescence was detected and analyzed using an Fluoroskan Ascent microplate reader (Thermo Scientific, Waltham, MA, USA) at maximum excitation and emission spectra of 485 and 535 nm, respectively. The fold change of ROS between dark- and BL-treated cells was calculated. The same experiment was performed in the presence of 100 μM rotenone, 500 μM 2,4-dinitrophenol (DNP), 50 μM flufenamic acid, 50 μM etofenamate, and 20 mM malonate.

### Construction of *V. cholerae* deletion mutants

We used the PCR method described in ref. 54 to generate an in-frame deletion mutant of each of the target genes listed in Table 1 using the primers listed in Supplementary Table S9. Briefly, P1 primer sets for each target gene were used to amplify approximately 500-bp 5′ flanking sequences (including ~150 bp of the coding sequence) and P2 primer sets were used to amplify approximately 500-bp of the 3′ regions (including ~150 bp of the coding sequence). The two PCR products were joined using the splicing overlap extension technique, and the resulting PCR product that lacked most of the internal coding sequence of the gene was digested with SacI and XbaI and cloned into the similarly digested pGP704-*sacB*28 suicide vector^55^. The *E. coli* SM10λpir strain^56^ was used to maintain the pGP704-*sacB*28 plasmid carrying the deletion construct. Biparental mating was carried out between recipient wild-type *V. cholerae*^8^ and the *E. coli* SM10λpir strain harboring the deletion construct. Ampicillin-resistant *V. cholerae* strains resulting from a single homologous recombination were identified, grown without ampicillin, and then subjected to 10% sucrose selection. Strains with the properties of a double recombination event (ampicillin-sensitive and sucrose-resistant) were identified and further analyzed by PCR with the flanking primers (Supplementary Table S9) to confirm that the gene was successfully deleted.

### Preparation of cDNA library for RNA-seq

The cDNA library preparation method was carried out as previously described^57^ and summarized in Supplementary Fig. S6. Briefly, total RNA was extracted from dark-grown and BL-exposed cell cultures (500 μl of culture with OD_600_ = 1.0) using TRIzol reagent (Invitrogen, Carlsbad, CA, USA) according to the manufacturer’s instructions. After quality and quantity measurements using BioAnalyzer 2100 (Agilent, Palo Alto, CA, USA), the RNA was treated with RNase-free DNase I (Thermo Scientific, Waltham, MA, USA) at a concentration of 1 U/μg to remove residual genomic DNA. To remove the rRNA, 2.5 μg total RNA was treated with a Ribo-Zero bacterial rRNA removal kit (Illumina, San Diego, CA, USA) following the manufacturer’s instructions. The rRNA-depleted RNA pellets obtained after the ethanol precipitation step were re-suspended in 10 μl RNase free water, and then samples were fragmented using a TruSeq mRNA sample preparation kit (Illumina, USA). The cleaved short RNA fragments were used for first-strand cDNA synthesis using random hexamer primers, and then the second strand was synthesized using DNA polymerase I and RNase H. The double-stranded cDNAs were purified with AMPure XP beads (Beckman Coulter, Brea, CA, USA) and eluted with resuspension buffer followed by 3′-end adenine nucleotide addition. Finally, sequencing adaptors were ligated to the fragments and cDNA fragments were enriched by PCR amplification. Enriched cDNA libraries were used for cluster generation and sequencing. Paired-end sequencing of the cDNA libraries (dark- and BL-treated) of wild-type and knockout *V. cholerae* cells was performed in duplicate (biological replicates) using the Illumina MiSeq sequencing platform (Illumina).

### Transcriptome analysis

All sequence data were at 2 × 75-bp length. The high-quality reads were saved in fastq files and deposited at the GEO database at NCBI under the accession number: GSE79911 (http://www.ncbi.nlm.nih.gov/geo/query/acc.cgi?token=mhwraiymxvsxnkn&acc=GSE79911). The processing of fluorescent images into sequences, base calling, and quality value calculations were performed by the Illumina data processing pipeline (v1.5). For quality control, reads with adaptor contamination and low-quality bases (quality score < Q30) were removed using Trimmomatic (v0.35). All the downstream analyses were based on high-quality, clean data.

The RNA-seq data were analyzed with Rockhopper^18^. A summary of the analytical methods is provided below. Reads were aligned to the *V. cholerae* O1 biovar El Tor str. N16961 genome with the following parameters: minimum seed length 0.33, allowed mismatches 5%. After aligning the sequencing reads to the genome, reads from each experiment were normalized by upper quartile normalization^58^. A Pearson’s correlation analysis was conducted to obtain the transcript-level R^2^ value between replicates. We used the false discovery rate (*FDR)* < 0.01 obtained from Rockhopper and the absolute value of |log_2_ fold change| ≥ 1 as the threshold to judge significant differences in gene expression.

To annotate the hypothetical DEGs, we used the custom pipeline Annocript^21^ (https://github.com/frankMusacchia/Annocript/tree/master/GUIDE). We used Swiss-Prot (SP) and UniRef90^22^ (version: August 2015) databases for blastp searches with the following parameters: word_size = 4; e-value = 10^−5^; num_descriptions = 5; num_alignments = 5; threshold = 18. For each sequence, the best hit was chosen. The domain composition of 3826 *V. cholerae* proteins in the Conserved Domains Database^59^ (https://www.ncbi.nlm.nih.gov/Structure/bwrpsb/bwrpsb.cgi) was determined by Rpsblast with the automatic search mode and e-value ≤ 10^−5^.

### GO term and KEGG pathway enrichment analyses

To functionally categorize the DEGs, a GO term enrichment analysis was conducted using the PANTHER classification system^19^ (http://www.pantherdb.org/) with Bonferroni multiple correction testing. After GO term functional enrichment analysis, the correlations were analyzed to construct a network using the BiNGO plug-in (v2.44) (http://apps.cytoscape.org/apps/bingo^20^) in Cytoscape (v2.8.3) (http://www.cytoscape.org/)^60^. KOBAS (v2.0) (http://kobas.cbi.pku.edu.cn) was used to test for the statistical enrichment of differentially expressed genes in KEGG pathways. Fisher’s exact test was used to calculate *p*-values, and pathways with a *p*-value < 0.05 were designated as being significantly enriched in DEGs.

### Hierarchical clustering and heatmap generation

For the heatmap display, the expression values of DEGs in dark and BL conditions were log_2_ transformed, and hierarchical clustering was performed on genes and arrays using the Euclidean distance similarity metric with the centroid linkage clustering method. Heatmaps were generated and visualized using Cluster3.0 (v1.52) and Java Treeview (v1.1.6r4), respectively.

### Validation of RNA-seq results using qRT-PCR

Twenty-one representative BL-responsive genes (VC0837, VC0943, VC1118, VC1248, VC1263, VC1359, VC1392, VC1484, VC1570, VC1643, VC1814, VC1922, VC2088, VC2301, VCA0055, VCA0615, VCA0782, VCA0798, VCA0809, VCA0957, VCA1087) were selected for validation by qRT-PCR. Total RNA was extracted using TRIzol reagent (Invitrogen, Carlsbad, CA, USA) according to the manufacturer’s instructions. After DNase I treatment, 1 μg total RNA was used for first-strand cDNA synthesis using random hexamer oligos. The qRT-PCRs were performed with Luminaris HiGreen qRT-PCR Master Mix (Thermo Scientific, Waltham, MA, USA) on the CFX Connect™ Real-Time PCR Detection System (Bio-rad, Hercules, CA, USA) using gene-specific primers (Supplementary Table S10), with *gapdh* (VC2000) as the internal reference gene. The amplification program was as follows: 95 °C for 10 min; 40 cycles of 95 °C for 2 s, 56 °C for 10 s, and 72 °C for 10 s, followed by a thermal denaturing step to generate the melting curves. All reactions were performed in biological triplicate (each triplicate with two technical replicates), and the results were expressed relative to the transcript level of *gapdh* in each sample using the 2^−ΔΔCT^ method^61^. The mRNA expression data were analyzed using IBM SPSS ver. 20.0 (SPSS Inc., Chicago, IL, USA). All relative mRNA expression data are presented as mean ± S.D. (*n* = 6).

## Additional Information

**How to cite this article**: Tardu, M. *et al*. MerR and ChrR mediate blue light induced photo-oxidative stress response at the transcriptional level in *Vibrio cholerae. Sci. Rep.* **7**, 40817; doi: 10.1038/srep40817 (2017).

**Publisher's note:** Springer Nature remains neutral with regard to jurisdictional claims in published maps and institutional affiliations.

## Supplementary Material

Supplementary Information

Supplementary Table S1

Supplementary Table S2

Supplementary Table S3

Supplementary Table S4

Supplementary Table S5

Supplementary Table S6

Supplementary Table S7

Supplementary Table S8

Supplementary Table S9

Supplementary Table S10

## Figures and Tables

**Table 1:** Bacterial strains and plasmids used in this study.

Strain or plasmid	Relevant genotype	Source
*E. coli* strains		
SM10-λ*pir*	Km^R^, thi^−1^, thr, leu, tonA, lacY, supE, recA::RP4-2-Tc::Mu, pir	62
*V. cholerae* strains		
MT_VC_0001	*Vibrio cholerae* O1 biovar El Tor N16961, wild-type, Str^r^	^63^
MT_VC_0002	ΔVCA0056 (*MerR*)	This study
MT_VC_0003	ΔVCA0057 (*phr*), Kan^r^	55
MT_VC_0004	ΔVC1392 (*cry2*), Tet^r^	This study
MT_VC_0005	ΔVC1814 (*cry1*)	This study
MT_VC_0006	ΔVC2301 (C*hrR*)	This study
MT_VC_0007	ΔVC2302 (*SigmaE)*	This study
MT_VC_0453	ΔVC1392ΔVC1814 ΔVCA0057 (*cry1,cry2,phr),* Kan^r^, Tet^r^	This study
MT_VC_0062	ΔVC2301 ΔVCA0056 (*ChrR, MerR)*	This study
Plasmids		
pGP704-*sacB*28	pGP704 derivative, *mob*/*oriT sacB*, Amp^r^	55
pMT-0003	pGP704-*sacB28*::ΔVC1392, Amp^r^	This study
pMT-0004	pGP704-*sacB28*::ΔVC1814, Amp^r^	This study
pMT-0005	pGP704-*sacB28*::ΔVC2301, Amp^r^	This study
pMT-0006	pGP704-*sacB28*::ΔVC2302, Amp^r^	This study
pMT-0007	pGP704-*sacB28*::ΔVCA0056, Ampr	This study

**Table 2:** Significantly up-(UR) and down-regulated (DR) KEGG pathways by blue light in wild-type cells (p < 0.05).

KEGG pathway name	KEGG pathway ID	# of DEGs	# of total genes	*p*-value	Regulation
Citrate cycle (TCA cycle)	vch00020	9	24	5.97E-06	UR
Butanoate metabolism	vch00650	7	27	2.48E-03	UR
Geraniol degradation	vch00281	3	5	8.20E-03	UR
C5-Branched dibasic acid metabolism	vch00660	4	11	8.42E-03	UR
Propanoate metabolism	vch00640	5	19	9.87E-03	UR
Lysine degradation	vch00310	3	7	1.61E-02	UR
Oxidative phosphorylation	vch00190	6	34	2.37E-02	UR
Carbon metabolism	vch01200	11	90	2.65E-02	UR
Fatty acid degradation	vch00071	3	9	2.72E-02	UR
Biosynthesis of secondary metabolites	vch01110	23	248	3.07E-02	UR
Bacterial chemotaxis	vch02030	10	67	8.79E-03	DR
Two-component system	vch02020	9	146	3.23E-04	DR
ABC transporters	vch02010	7	126	2.88E-03	DR
Glyoxylate and dicarboxylate metabolism	vch00630	2	22	4.79E-02	DR

**Table 3:** Significantly up-(UR) and down-regulated (DR) KEGG pathways in Δ*ChrR* mutants under blue light (*p* < 0.05).

KEGG pathway name	KEGG pathway ID	# of DEGs	# of total genes	*p*-value	Regulation
*Geraniol degradation	vch00281	3	5	4.91E-04	UR
*Fatty acid degradation	vch00071	3	9	1.81E-03	UR
*Propanoate metabolism	vch00640	3	19	1.09E-02	UR
Fatty acid metabolism	vch01212	3	19	1.09E-02	UR
Valine, leucine and isoleucine degradation	vch00280	2	9	2.21E-02	UR
*Butanoate metabolism	vch00650	3	27	2.54E-02	UR
*C5-Branched dibasic acid metabolism	vch00660	2	11	3.05E-02	UR
Histidine metabolism	Vch00340	2	14	4.44E-02	UR
*Bacterial chemotaxis	vch02030	8	67	2.20E-11	DR
*Two-component system	vch02020	7	146	6.32E-11	DR
*ABC transporters	vch02010	6	126	2.50E-04	DR

^*^Indicates affected pathways in wild-type cells by blue light exposure.
