# Supplementary material for: MerR and ChrR mediate blue light induced photo-oxidative stress response at the transcriptional level in Vibrio cholerae
Source: Sci Rep. 2017 Jan 18;7:40817. doi: 10.1038/srep40817 (PMC5241685; doi:10.1038/srep40817)
Supplement: Supplementary Information [file srep40817-s1.pdf]

MerR and ChrR mediate blue light induced photo-oxidative stress response at the transcriptional level in *Vibrio cholerae*

Mehmet Tardu[1]

Email: mtardu@ku.edu.tr

Selma Bulut[2]

Email: sbulut@ku.edu.tr

Ibrahim Halil Kavakli[1,2,3]*

Email: hkavakli@ku.edu.tr

[1]Computational Science and Engineering, Koc University, Rumeli Feneri Yolu, Sariyer, Istanbul, Turkey

[2]Chemical and Biological Engineering, Koc University, Rumeli Feneri Yolu, Sariyer, Istanbul, Turkey

[3]Molecular Biology and Genetics, Koc University, Rumeli Feneri Yolu, Sariyer, Istanbul, Turkey

*Corresponding author

Tel:+90 212 338 1708

Fax: :+90 212 338 1548

E-mail:hkavakli@ku.edu.tr

Supplementary Figure Legends

Supplementary Figure S1: Comparison of gene transcript levels between replicates of dark- and blue-light-treated samples. Correlation plots for transcript-level RPKM values between replicates of wild-type, *ΔChrR*, *ΔChrRΔMerR* and *Δcry1Δcry2Δphr* knockout cells treated with dark or BL. Pearson R^2 values for correlation are included in plots.

Supplementary Figure S2: Validation of RNA-seq data by qRT-PCR. RNA-seq results of selected 21 DEGs (Set2) in response to blue light were compared with qRT-PCR results. Blue bars with standard errors represent relative transcript levels determined by qRT-PCR (with respect to dark treated cells, $\log_2$ fold) from three independent biological replicates. Green bars represent transcript levels determined using RNA-seq.

Supplementary Figure S3: Effect of blue light (BL) and red light (RL) on the expression levels of differentially expressed genes (DEGs)**.** Transcript levels of selected 21 DEGs (Set2) were determined after exposure of wild-type cells to BL and RL. Blue (BL exposure) and red (RL exposure) bars with standard errors represent relative mRNA expression levels with respect to dark conditions ($\log_2$ fold) determined by qRT-PCR from three independent biological replicates. $n = 6$, * $p < 0.05$, Student's t-test.

Supplementary Figure S4: Reactive oxygen species (ROS) accumulation in wild-type (WT), *ΔChrR* and *ΔChrRΔMerR* knock out cells after blue light (BL) exposure. Cells were exposed to BL (50 µmoles $m^{-2}s^{-1}$) for 45 min, then samples were collected and total ROS amount was measured using 2′,7′ dichlorofluorescein. Fold change was calculated between dark- and BL-treated samples at indicated times.

Supplementary Figure S5: Flow-chart of ROS measurement protocol**.**

Supplementary Figure S6: Flow-chart of RNA-seq analysis**.**

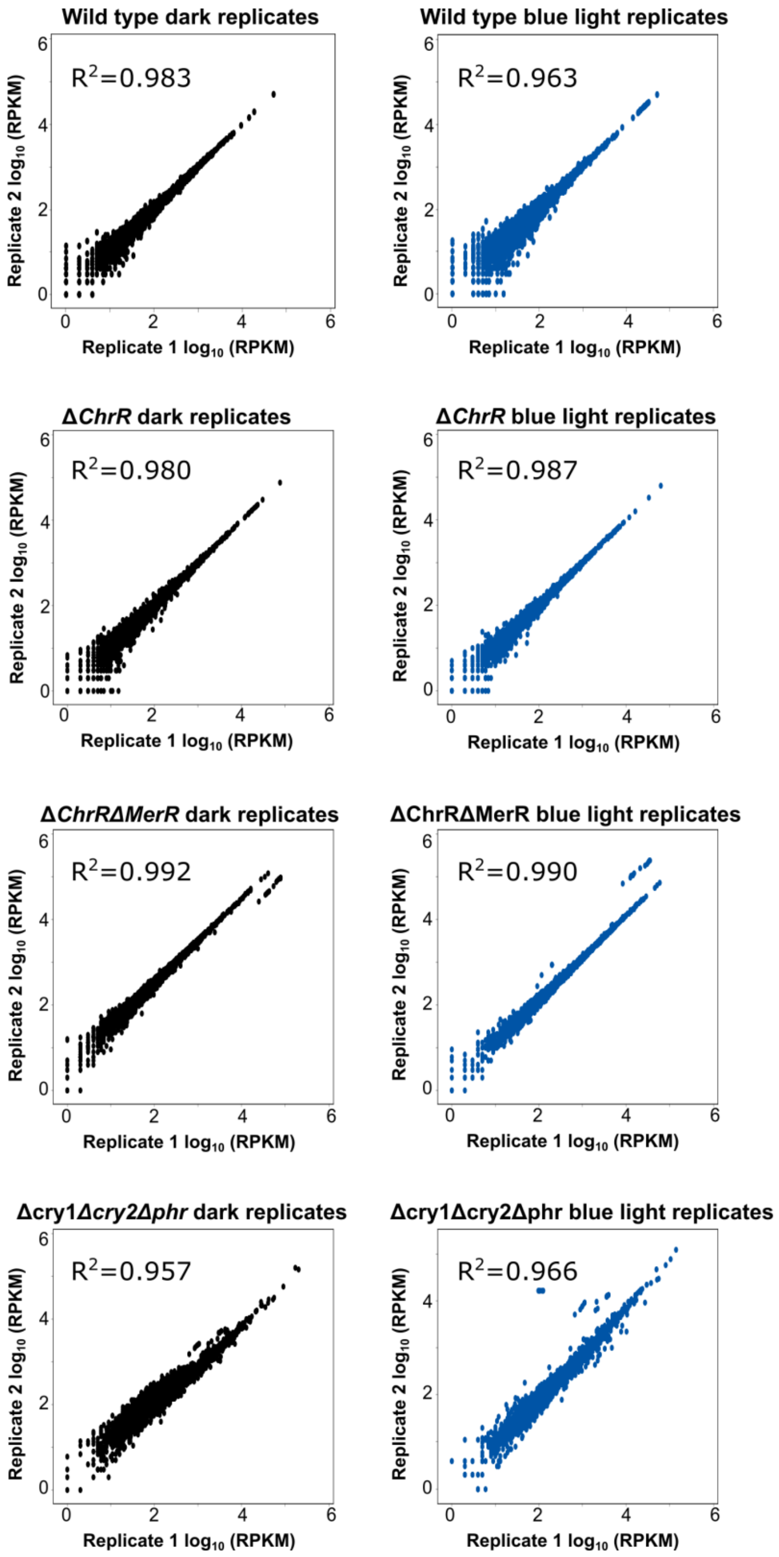

Supplementary Figure S1

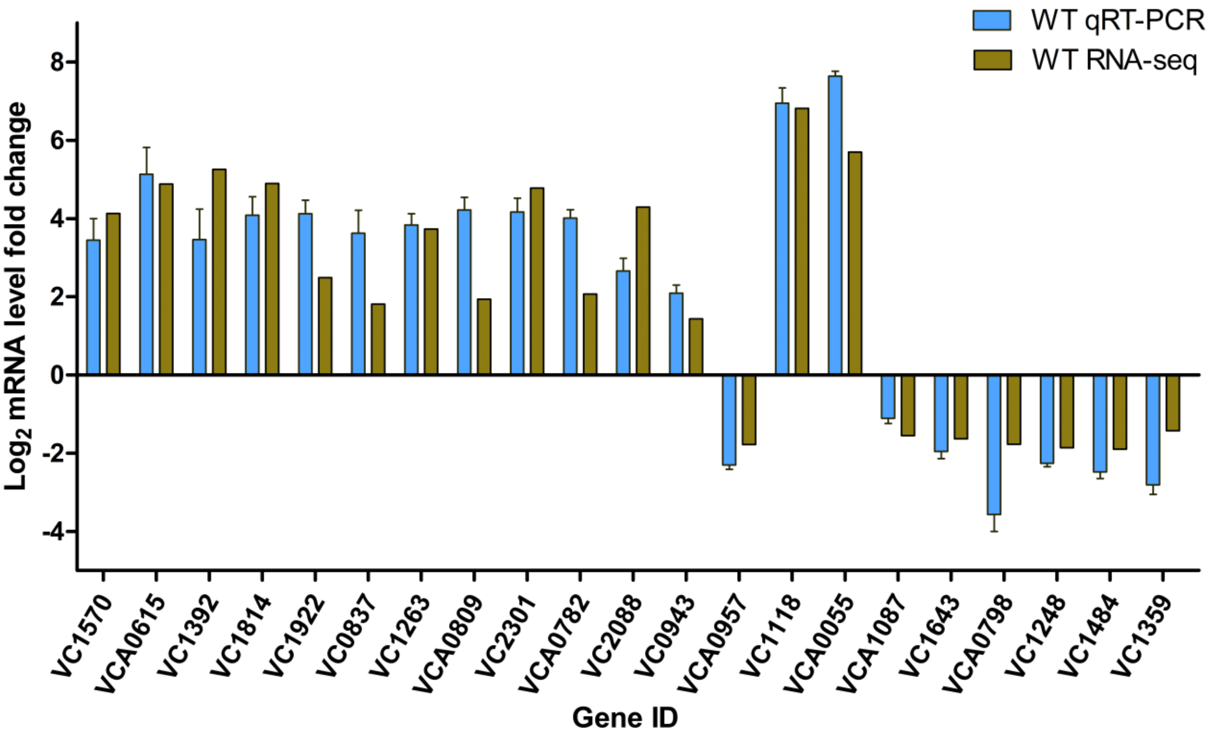

Supplementary Figure S2

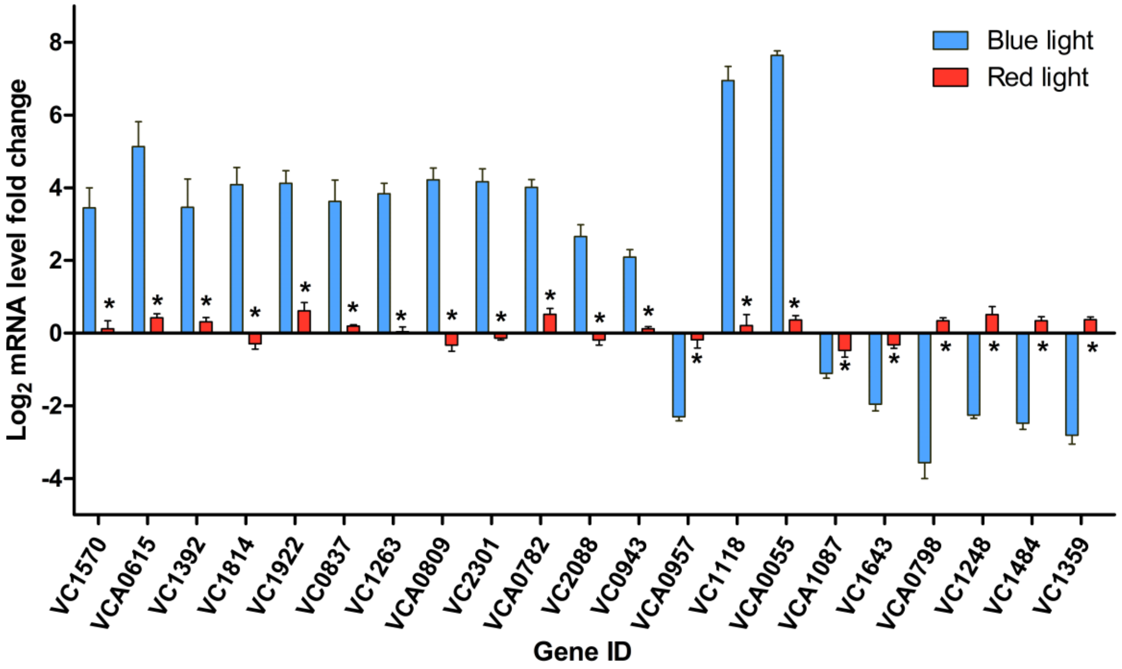

Supplementary Figure S3

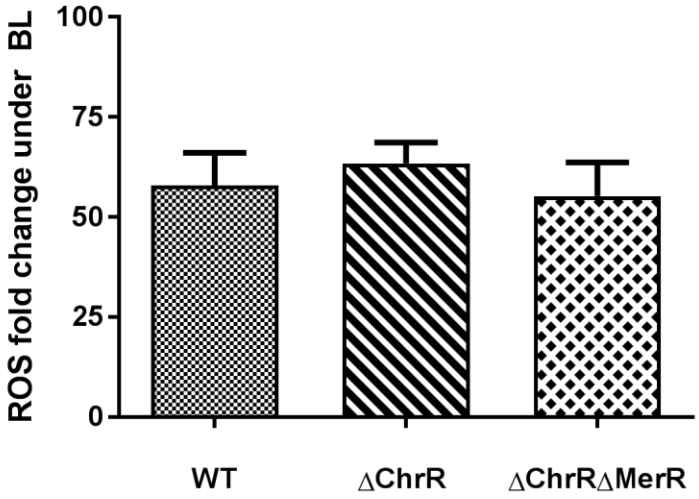

Supplementary Figure S4

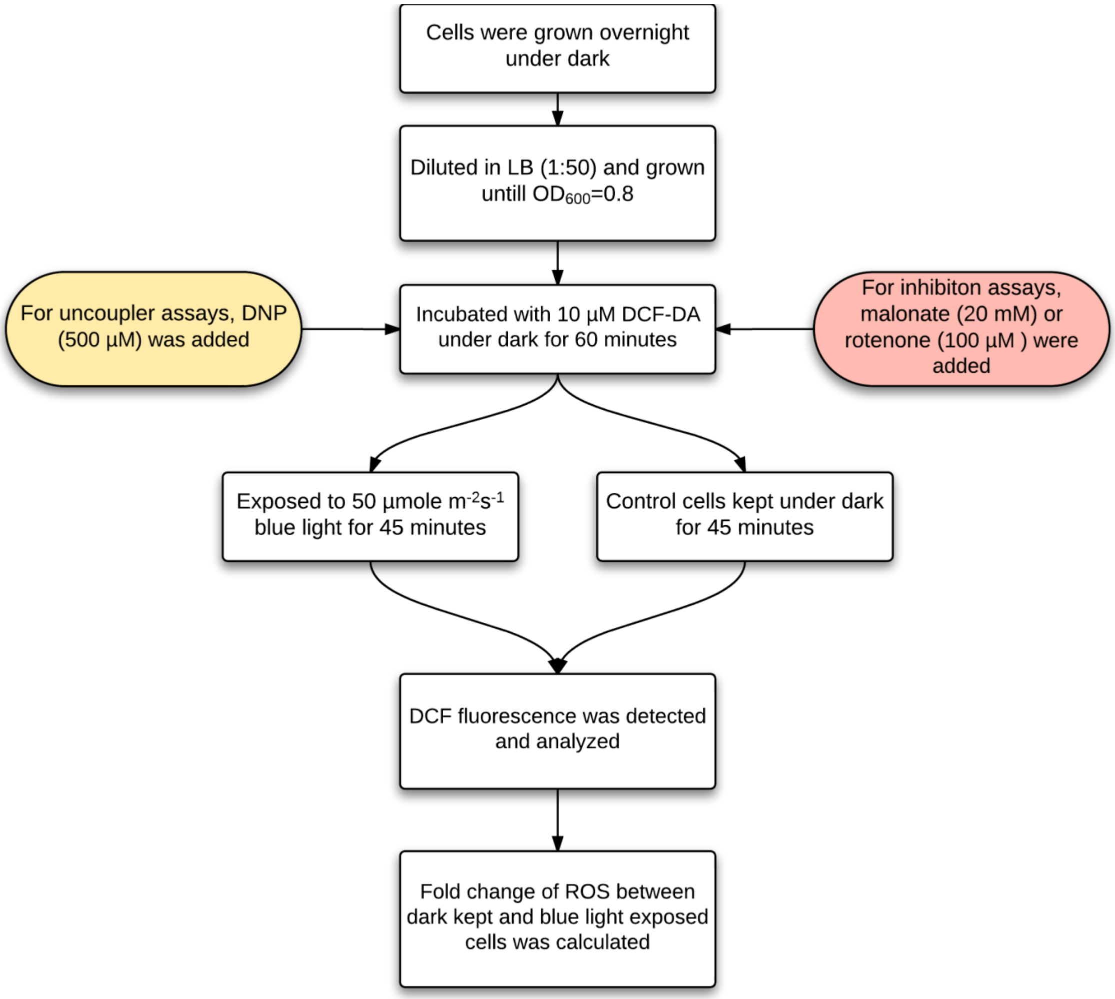

Supplementary Figure S5

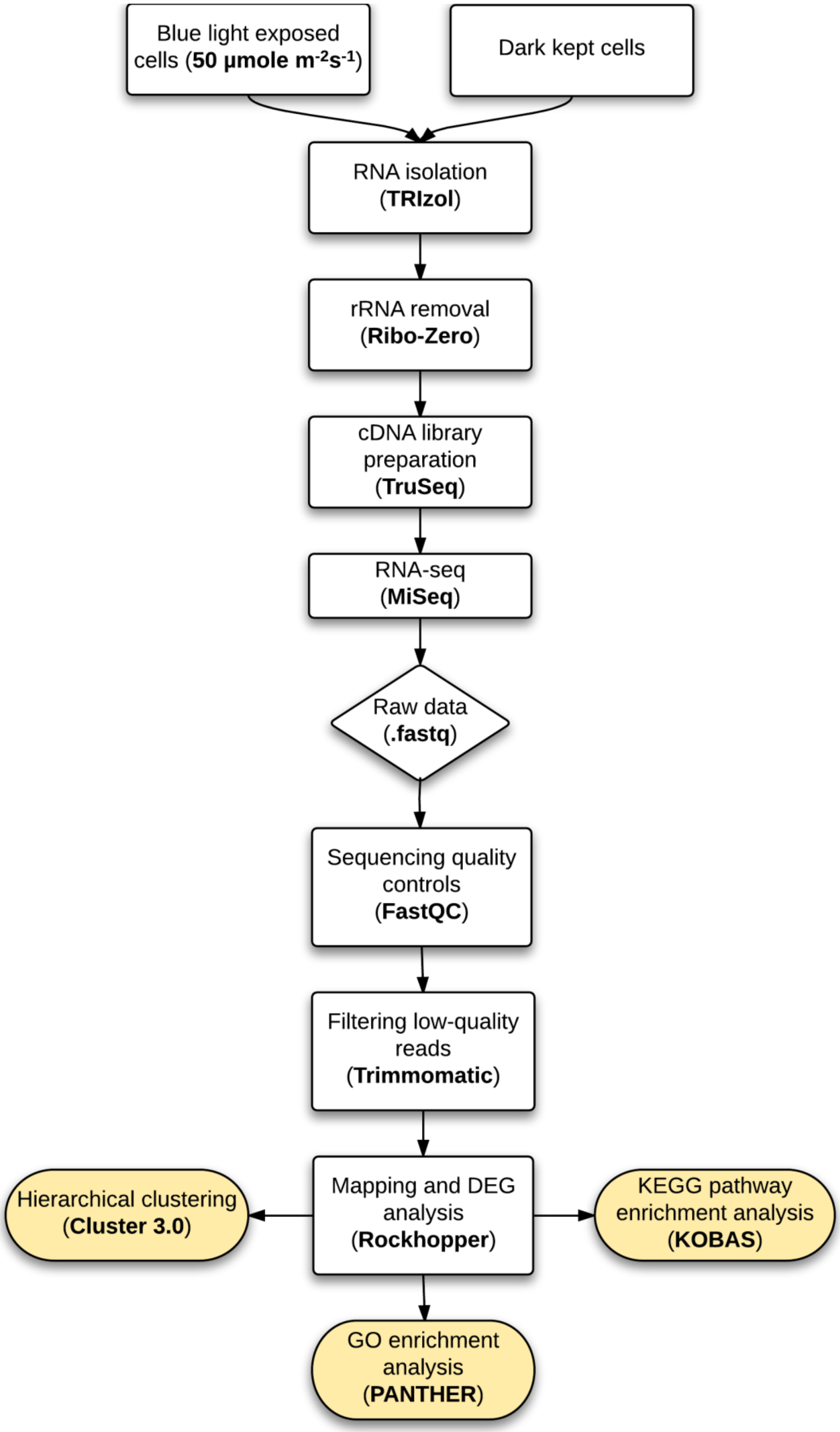

Supplementary Figure S6
